# Supplementary material for: Viromes of Tabanids from Russia
Source: Viruses. 2023 Nov 30;15(12):2368. doi: 10.3390/v15122368 (PMC10748123; doi:10.3390/v15122368)
Supplement: Supplementary file 1 [file viruses-15-02368-s001.zip › viruses-2718474-supplementary.pdf]

Table S1. Oligonucleotides used for PCR-detection of the novel viruses.

Pool	Virus	Oligonucleotide name	Oligonucleotide sequence	Product length	Temperature
1	Xanka Hybomitra Negev-like virus	NegevL-p1_F	GATGCGTTAAAAGAAGCGAA	310	50
		Negev_p1_P	(FAM)-ACCGACAAGCCAGTGATACCTGACA-(BHQ-1)		
		NegevL-p1_R	TTGGACAAAACACGGAATTG		
	Razdolnyj Hybomitra Phasma-like virus	Orto_p1_F	TGTTGTTGCTAATGTTCCAC	308	50
		Orto_p1_R	ATTCTGATGATTCCTGGGTG		
2	Kamenushka Hybomitra Narna-like virus	Narn_p2_F	CGTTAATCATTTGGCGTTGA	399	50
		Narn_p2_P	(FAM)-GCGCACTGAGGACAGTGGCC-(BHQ-1)		
		Narn_p2_R	CCATTTTTGAGGGTTGACAG		
	Volxa Hybomitra Toti-like virus	Toti_p2_F	AACTCGACGATGAATGTGAA	358	50
		Toti_p2_P	(FAM)-GCAGCTTGGTATCTGATGGCGCT-(BHQ-1)		
		Toti_p2_R	GAGAAGTGACTCGTAAGGAC		
	Komarovka Hybomitra Solemo-like virus	Sole_p2_F	CGTGCATTCCCGCTATTTGT	362	55
		Sole_p2_P	(FAM)-GCTCCCACTGTGCCTTGAC-(BHQ-1)		
		Sole_p2_R	AGTGATGCCAGCCCATGTTT		
	Barsukovka Hybomitra Ifla-like virus	Ifla_p2_F	TTTGTATTGATCCAGGTGCT	363	52
		Ifla_p2_P	(FAM)-ACTTTGAGACCACCCCGCCA-(BHQ-1)		
		Ifla_p2_R	TTTCTGCTATGCCTAAAGGT		
3	Big rock Hybomitra Ifla-like virus	C30_p3_F	TTCTTGCTGCACTAGTATTGA	252	50
			TGGGTCTGTGTTGGACAATAACACCCA		

		C30_p3_R	GAATTGACTGATGATGCTTCT		
6	Medvezhye Haematopota Flavi-like virus	LFla_p6_F	CCGAAAGTCAAGAAGAATGC	286	50
		LFla_p6_R	CTGGATGGTATATGCGTTGA		
	Melisia Haematopota Negev-like virus	NegevL_p6_F	CGTATGTTTGTCATTCGCAA	261	50
		NegevL_p6_P	(FAM)-CCATAGGCAGGGCAGTCACA-(BHQ-1)		
		NegevL_p6_R	CAGACCAAAAGGACTCATCA		
	Medvezhye Haematopota Xinmo-like virus	Anphe_p6_F	TTGTTATTATCCCAGGTGCC	367	50
		Anphe_p6_R	CGTCCTCTTGGATCTGTTAG		
	Medvezhye pound Haematopota Permuto-like virus	Permu_p6_F	CCCAACGCATATCCTCATTA	283	50
		Permu_p6_P	(FAM)-TGCCACGGGATGTTTCAACC-(BHQ-1)		
		Permu_p6_R	GAGCGGACAAATATACCCAT		
	Polka Haematopota Nora-like virus	C191_p6_F	TTTTGGAGGATTTGTGAATGC	254	50
		C191_p6_P	(FAM)-GATCCTCACAGCCCGGCGTG-(BHQ-1)		
		C191_p6_R	CCTGAATCTTTCTGGCCTTAT		
	Polka Haematopota Xinmo-like virus	C223_p6_F	TACCAAGTAGGCAACATACC	270	50
		C223_p6_R	TGTTTCAGACATGAACTGGT		
7	Medvezhye Chrysops Negev-like virus	NegevL_p7_F	CATTGACCACATCTTGCATC	400	52
		NegevL_p7_P	(FAM)-GGCATGTGAAGTGGTAATGGAATC-(BHQ-1)		
		NegevL_p7_R	ACTAAGGAGGGTCTAGTGTC		
	Big Soux River virus (strain Medveje)	BSR_p7_F	TTTAATATGCAACCACGGGA	274	50
		BSR_p7_P	(FAM)-TGCGCACTCGTCCCGAATGA-(BHQ-1)		

		BSR_p7_R	TGGTAGAACAGGATTGTGTG		
	Medvezhye Chrysops Solemo-like virus	Sol1_p7_F	TCTTTCTTCAAGATCGGCAA	326	50
		Sol1_p7_P	(FAM)-TCTGGGCGGCAGCAACTATG-(BHQ-1)		
		Sol1_p7_R	GGAGAGTCAGCATTGGTTTA		
	Melisia Chrysops Solemo-like virus	Sol2_p7_F	ATGATCAAACCGTGTTGAGA	358	50
		Sol2_p7_P	(FAM)-AACCATTTGCCACAAGATCTGTAA-(BHQ-1)		
		Sol2_p7_R	CAGTGATCAGAGCCTTAGAC		
	Polka Chrysops Solemo-like virus	Sol3_p7_F	AAAGTAAAACCAGGCGTTTG	268	50
		Sol3_p7_P	(FAM)-AACTCGAACGATTGATGCGGT-(BHQ-1)		
		Sol3_p7_R	AACTCGATGTATTCTGGCTC		
	Medvezhye Chrysops Narna-like virus	Nar1_p7_F	TTTTCTTCGACATATGGGCA	255	50
		Nar1_p7_R	GCTTTGATATCAGATGCTGC		
	Medvezhye Chrysops Narna-like virus 2	Nar2_p7_F	ACAGTGCGAAGTTGTTATCT	327	50
		Nar2_p7_P	(FAM)-CAAATGGCTCGGCTTGGTAGATCG-(BHQ-1)		
		Nar2_p7_R	AGATCGGATAGCTTCGTCTA		
	Medvezhye Chrysops Solinvi-like virus	Soli_p7_F	ACATTGCCTTTTCCATTGTG	335	50
		Soli_p7_R	AAAAGTTAGGCGTGACAGAT		
	Medvezhye Chrysops Rhabdo-like virus	Rha_p7_F	AGAGGCTCTCACTGACAATA	292	50
		Rha_p7_P	(FAM)-TGGCTGACGGATCTGAGATCCCT-(BHQ-1)		
		Rha_p7_R	CCCTCCTAAATGTCCTTTCC		

8	Medvezhye Chrysops Ifla-like virus	Ifla_p8_F	CATAAGAAGCATAACGACGC	350	50
		Ifla_p8_P	(FAM)-ACGATCTCCCGATTTCATCCATTCTGA-(BHQ-1)		
		Ifla_p8_R	TGTGATGTGTCCTTACTTCG		
	Medvezhye Chrysops Toti-like virus	Toti_p8_F	TGGTAACATCCAGTTCACAG	387	50
		Toti_p8_P	(FAM)-TGCCGCTATGGGGGAGGCAT-(BHQ-1)		
		Toti_p8_R	TTTTCCGCCGTTATAGGTAG		
	Istie Chrysops Solemo-like virus	Sole_p8_F	ATTCCAATTCCGAGTCAACA	268	50
		Sole_p8_P	(FAM)-AGTTGGGTAATTTCGGGGTCTTGGA-(BHQ-1)		
		Sole_p8_R	CGACCCTCCAAAAGTAAAGA		
	Medvezhye Chrysops Xinmo-like virus	Rha_p8_F	GTTCAAGCTTAGGGGTTACA	255	50
		Rha_p8_P	(FAM)-TCACGCACATGGTACAGATGCAA-(BHQ-1)		
		Rha p8_R	TATGTGATGCCAGTACGTTT		
	Medvezhye Chrysops Phasma-like virus	Orto_p8_F	GCATGCACTCATAGATGTTG	260	50
		Orto_p8_R	GTGACTGGATTATCAACGGA		
9	Medvezhye Tabanus Phasma-like virus	Orto_p9_F	ACTACAAGTTTAGGTGCCAG	370	50
		Orto_p9_R	ATGATGTGTTAACTGCTGGT		
	Medvezhye Tabanus Chu-like virus	Rha_p9_F	TCACTTAAAGTTGCGGATGA	334	50
		Rha_p9_R	TTACAACTTATGGACACGCA		
	Medvezhye Tabanus Toti-like virus	Toti_p9_F	AGTCTTAGCCTATACAGCCA	353	50
		Toti_p9_P	(FAM)-TCCTCCATGCCCGGCTTGAGT-(BHQ-1)		
		Toti_p9_R	TGATTAGGCCACATGTGAAA		

Table S2. Oligonucleotides used for internal control Poliovirus RT-qPCR.

Oligonucleotide	Sequence
PVR1	5’-CGAACGTGATCCTGAGTGTT-3’
PVP1	(ROX)-TTGATTCATGAATTTCCTTCATTGGCA-(BHQ-2)
PVL1	5’- GGCAGACGAGAAATACCCAT- 3’

Table S3. Characteristics of viruses with complete and partial coding sequences.

Pool	Virus	Presence in the pool (%)	Comparison protein	Closest relative	Q cover	Identity
1	Xanka Hybomitra Negev-like virus	0.01	ORF1 (RdRp)	[URQ09136.1] Halyomorpha halys negev-like virus 1	61%	42.2%
2	Kamenushka Hybomitra Narna-like virus	<0.01%	RdRp	[QVG74774.1] Riboviria sp.	89%	52.5%
	Volxa Hybomitra Toti-like virus	0.01	RdRp	[UPT53761.1] Bactrocera zonata toti-like virus	99%	41.1%
	Komarovka Hybomitra Solemo-like virus	<0.01%	RdRp	[UJG28005.1] Ulaatai Melophagus solemo-like virus	75%	63.6%
			CP	[APG75767.1] Hubei sobemo-like virus 48	93%	52.7%
6	Medvezhye Haematopota Flavi-like virus	0.29	Polyprotein	[QTJ63573.1] Orthopteran flavi-related virus	15%	41.7%
	Melisia Haematopota Negev-like virus	0.23	ORF1 (RdRp)	[URQ09136.1] Halyomorpha halys negev-like virus 1	61%	39.2%
	Medvezhye Haematopota Xinmo-like virus	0.18	RpRp	[YP_009337182.1] Hubei diptera virus 11	98%	35.9%
	Medvezhye pound Haematopota Permuto-like virus	0.03	Polyprotein	[WKV34304.1] Riboviria sp.	61%	35.8%
7	Medvezhye Chrysops Negev-like virus	1.08	ORF1 (RdRp)	[UPT53679.1] Zeugodacus tau negev-like virus	73%	44.3%
	Big Soux River virus (strain Medvezhye)	0.48	ORF1	[ATI98938.1] Big Sioux River virus	99%	99.9%
	Medvezhye Chrysops Solemo-like virus	0.13	RdRp	[YP_009330098.1] Hubei sobemo-like virus 13	73%	51.1%
			CP	[YP_009342449.1] Wuhan house centipede virus 5	99%	37.7%
	Melisia Chrysops Solemo-like virus	0.28	RdRp	[UCS96389.1] Riboviria sp.	81%	55.7%
			CP	[APG75767.1] Hubei sobemo-like virus 48	86%	52.6%
	Polka Chrysops Solemo-like virus	0.05	RdRp	[QIJ70118.1] Jeffords solemo-like virus	95%	39.5%
			CP	[QHA33885.1] Atrato Sobemo-like virus 3	86%	35.4%
	Medvezhye Chrysops Narna-like virus	0.61	RdRp	[UHR49680.1] Sanya cydistomyia duplonotatay narnavirus 1	91%	51.2%
	Medvezhye Chrysops Narna-like virus 2	0.23	RdRp	[UHR49680.1] Sanya cydistomyia duplonotatay narnavirus 1	92%	51.4%
8	Medvezhye Chrysops Ifla-like virus	0.02	Polyprotein	[UQJ82152.1] Exitianus exitiosus virus 2	95%	34.5%
	Medvezhye Chrysops	0.03	RdRp	[YP_009333170.1] Hubei toti-	98%	60%

	Toti-like virus			like virus 19		
	Istie Chrysops Solemo-like virus	0.01	RdRp	[QXV86400.1] Solemoviridae sp.	68%	46.6%
			CP	[APG75767.1] Hubei sobemo-like virus 48	98%	42.2%
	Medvezhye Chrysops Xinmo-like virus	0.01	L	[YP_009337182.1]Hubei diptera virus 11	98%	39.2%

Table S4. Characteristics of genome fragments detected in the study.

Pool	Contig type and assigned name	Contig name	Closest relative	Protein	Q cover	Identity
1	**Orthophasma-like**<br><br>Razdolnyj Hybomitra Phasma-like virus	NODE_8_length_1310_	[QXV86665.1] Tibet bird virus 1	nucleocapsid	89%	42.8%
		NODE_19_length_530_	[QID77675.1] Pink bollworm virus 2	RdRp	100%	69.3%
		NODE_21_length_478_	[YP_009666959.1] Seattle Prectang virus	RdRp	100%	46%
		NODE_22_length_468_	[UHK03218] Hangzhou zicrona caerulea phasmavirus 1	RdRp	99%	53.3%
2	**Iflaviridae-like**<br><br>Barsukovka Hybomitra Ifla-like virus	NODE_11_length_1234_	[WEU50803.1] Chrysopa pallens	Polyprotein	93%	35.7%
		NODE_20_length_639_	[CAF1136435.1] Ceratitis capitata iflavirus 3	Polyprotein	96%	48.4%
		NODE_21_length_616_	[UJG27957.1] Khandagaity Melophagus ifla-like virus	Polyprotein	100%	36.9%
		NODE_24_length_577_	[WEU50803.1] Chrysopa pallens	Polyprotein	100%	31.8%
		NODE_27_length_467_	[QZZ63343.1] Leuven wasp-associated virus 3	Polyprotein	99%	46.1%
		NODE_28_length_466_	[UUG74229.1]XiangYun picorna-like virus 4	Polyprotein	79%	57.6%
		NODE_37_length_408_	[UQJ82152.1] Exitianus exitiosus virus 2	Polyprotein	100%	63%
3	**Iflaviridae-like**<br><br>Big rock Hybomitra Ifla-like virus	NODE_30_length_381_	[CAF1136435] Ceratitis capitata iflavirus 3	Polyprotein	98%	39.6%
6	**Nora virus-like**<br><br>Polka Haematopota Nora-like virus	NODE_191_length_616_	[ASM93992]Caledonia beadlet anemone Nora virus-like virus 1	ORF2	96%	42.9%
	**Xinoviridae-like**<br><br>Polka Haematopota Xinmo-like virus	NODE_223_length_580_	Hubei diptera virus 11	RdRp	97%	53.6%
7	**Solinviviridae-like**	NODE_115_length_1354_	[UHK03205.1] Hangzhou Solinvi-like virus 2	Polyprotein	99%	38%
		NODE_146_length_1248_	[UHK03205.1] Hangzhou	Polyprotein	99%	72%

	Medvezhye Chrysops Solinvi-like virus		Solinvi-like virus 2			
		NODE_267_length_903_	[UHK03205.1] Hangzhou Solinvi-like virus 2	Polyprotein	99%	68.6%
		NODE_312_length_849_	[UHK03205.1] Hangzhou Solinvi-like virus 2	Polyprotein	100%	75.5%
		NODE_382_length_787_	[UHK03205.1] Hangzhou Solinvi-like virus 2	Polyprotein	99%	74.2%
		NODE_574_length_664_	[UHK03205.1] Hangzhou Solinvi-like virus 2	Polyprotein	100%	75.1%
		NODE_617_length_640_	[UHK03205.1] Hangzhou Solinvi-like virus 2	Polyprotein	100%	46.5%
7	**Rhabdo-like** Medvezhye Chrysops Rhabdo-like virus	NODE_9_length_4303_	[AJG39162.1] Wuhan Fly Virus 3	L	99%	36.8%
7	**Rhabdo-like** Melisia Chrysops Rhabdo-like virus	NODE_120_length_1338_	[YP_009336837.1] Hubei lepidoptera virus 2	L	95%	46.6%
		NODE_12_NODE_108_	[UZC34538.1] jopcycgri virus 1	L	99%	50%
8	**Orthophasma-like** Medvezhye Chrysops Phasma-like virus	NODE_16_length_3720	[QXV86663.1]Tibet bird virus 1	RdRp	99%	52%
		NODE_43_length_2382_	[YP_010086190.1] Anopheles triannulatus orthophasmavirus	Glycoprotein	90%	29.4%
		NODE_163_length_1495_	[QXV86665.1]Tibet bird virus 1	N	69%	39.1%
		NODE_299_length_1184_	[QXV86663.1]Tibet bird virus 1	RdRp	99%	31.5%
8	Solinviviridae-like	NODE_2118_length_528_	[UHK03205.1]Hangzhou Solinvi-like virus 2	Polyprotein	67%	82.4%
		NODE_3267_length_436_	[UHK03205.1]Hangzhou Solinvi-like virus 2	Polyprotein	99%	70.8%
		NODE_4305_length_385_	[UHK03205.1]Hangzhou Solinvi-like virus 2	Polyprotein	99%	50.8%
9	**Orthophasma-like** Medvezhye Tabanus Phasma-like virus	NODE_10_length_1088_	[QXV86665.1] Tibet bird virus 1	N	97%	38.9%
		NODE_47_length_434_	[YP_009666982] Ganda bee virus	Glycoprotein	98%	37.9%
9	**Toti-like** Medvezhye Tabanus Toti-like virus	NODE_20_length_755_	[YP_009333170.1]Hubei toti-like virus 19	VP2	99%	67%
		NODE_24_length_700_	[YP_009333170.1]Hubei toti-like virus 19	VP2	96%	79.2%
		NODE_64_length_393_	[YP_009333170.1]Hubei toti-like virus 19	VP2	100%	67.7%
		NODE_69_length_384_	[YP_009333169.1]Hubei toti-like virus 19	VP1	100%	72.4%
9	**Chu-like** Medvezhye Tabanus Chu-like virus	NODE_19_length_798_	[YP_010798609.1] megalopteran chu-related virus 119	Glycoprotein	39%	52.8%
		NODE_22_length_739_	[YP_010798611.1] megalopteran chu-related virus 119	RdRp	97%	34.8%

		NODE_25_length_693_	[APG78769.1] Hubei chuvirus-like virus 4	RdRp	99%	48.3%
		NODE_35_length_520_	[YP_010798611.1] megalopteran chu-related virus 119	RdRp	100%	32.4%
		NODE_36_length_509_	[YP_010798611.1] megalopteran chu-related virus 119	RdRp	98%	54.5%

Table S5. Similarity estimation within tabanid negev-like viruses using discontiguous megablast on the full genome.

	Xanka Hybomitra Negev-like virus	Medvezhye Chrysops Negev-like virus
Xanka Hybomitra Negev-like virus	-	68.9% (51% Q cover)
Melisia Haematopota Negev-like virus	70.1% (57% Q cover)	71.2% (68% Q cover)

Table S6. Similarity estimation within tabanid xinmo-like sequences using L protein amino acid sequence.

	Medvezhye Haematopota Xinmo-like virus	NODE_223_length_580_
Medvezhye Haematopota Xinmo-like virus	-	57.2% (97% cover)
Medvezhye Chrysops Xinmo-like virus	60.1% (98% cover)	52.9% (98% cover)

Table S7. Similarity estimation within tabanid ifla-like sequences using polyprotein amino acid sequence.

Pool	Contig Name	Query cover to the GenBank Entry	Identity to the GenBank Entry	Query cover to Medvezhye Chrysops Ifla-like virus	Identity to the Medvezhye Chrysops Ifla-like virus
2	NODE_11_length_1234_	93%	35.7%	93%	50.0%
	NODE_20_length_639_	96%	48.4%	99%	59.3%
	NODE_21_length_616_	100%	36.9%	99%	43.4%
	NODE_24_length_577_	100%	31.8%	100%	35.0%
	NODE_27_length_467_	99%	46.1%	99%	54.5%
	NODE_28_length_466_	79%	57.6%	79%	59.6%
	NODE_37_length_408_	100%	63%	100%	72.5%
3	NODE_30_length_381_	98%	39.6%	98%	79.9%

Table S8. Similarity estimation within tabanid solinvi-like sequences using polyprotein amino acid sequence.

Pool 8 contig name	Pool 8 abundance	Pool 7 relative	Q cover	Identity
NODE_2118_length_528_	10 reads	NODE_146_length_1248_	35%	99.5%
NODE_3267_length_436_	3 reads	NODE_312_length_849_	93%	99.0%
NODE_4305_length_385_	5 reads	NODE_146_length_1248_	99%	96.9%

Table S9. Similarity estimation of pool 1 orthophasma-like contig with orthophasma-like sequences from pools 8 and 9 using amino acid sequences.

Pool 1 contig name	Pool 8 relative	Q cover	Identity	Pool 9 relative	Q cover	Identity
NODE_8_length_1310_	NODE_163_length_1495_	85%	46.3%	NODE_10_length_1088_	89%	62.6%
NODE_19_length_530_	NODE_16_length_3720_	100%	77.3%	no	-	-
NODE_21_length_478_	NODE_16_length_3720_	100%	56.2%	no	-	-
NODE_22_length_468_	NODE_16_length_3720_	99%	58.4%	no	-	-

Table S10. Similarity estimation of pool 9 orthophasma-like contig with orthophasma-like sequences from pools 8 and 1 using amino acid sequences.

Pool 9 contig name	Pool 8 relative	Q cover	Identity	Pool 1 relative	Q cover	Identity
NODE_10_length_1088_	NODE_163_length_1495_	86	45.9%	NODE_8_length_1310_	93%	62.6%
NODE_47_length_434_	NODE_43_length_2382_	98	42.0%	no	-	-

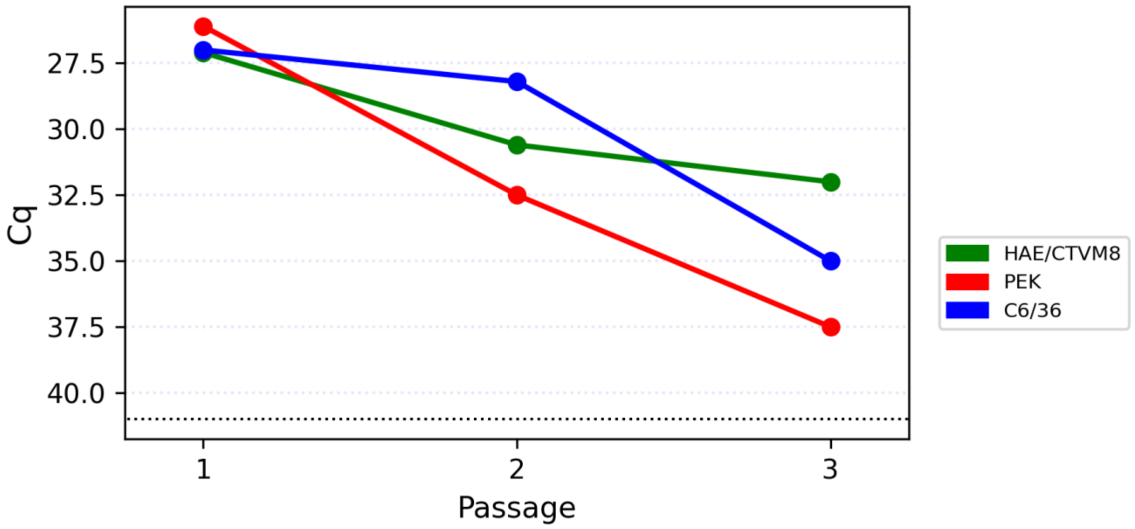

Figure S1. Dynamic of the real-time quantification cycle (Cq) during passages of the Xanka Hybomitra negev-like virus. Black dotted line represents qPCR detection limit (41 cycles).

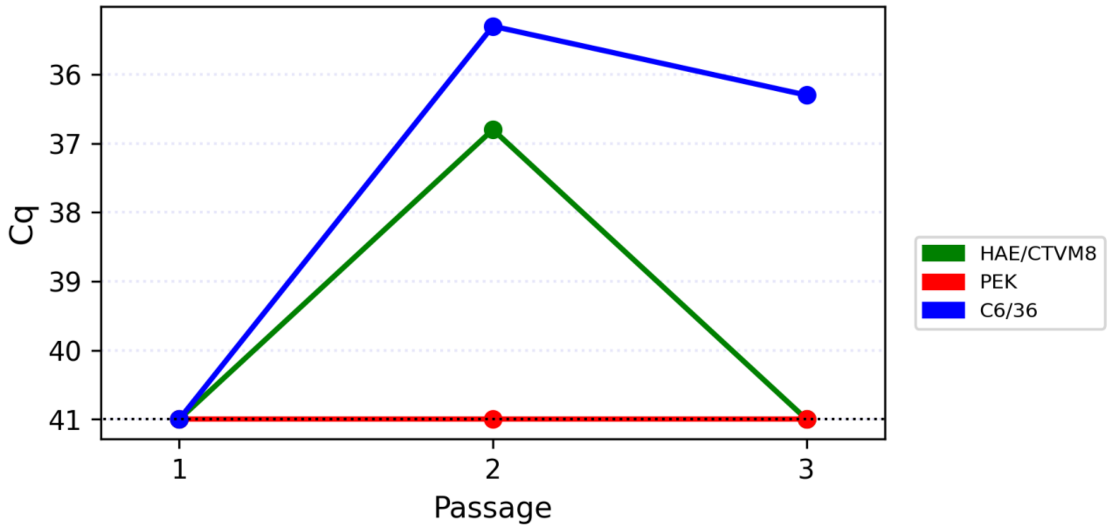

Figure S2. Dynamic of the real-time quantification cycle (Cq) during passages of the Volxa Hybomitra toti-like virus. Black dotted line represents qPCR detection limit (41 cycles).

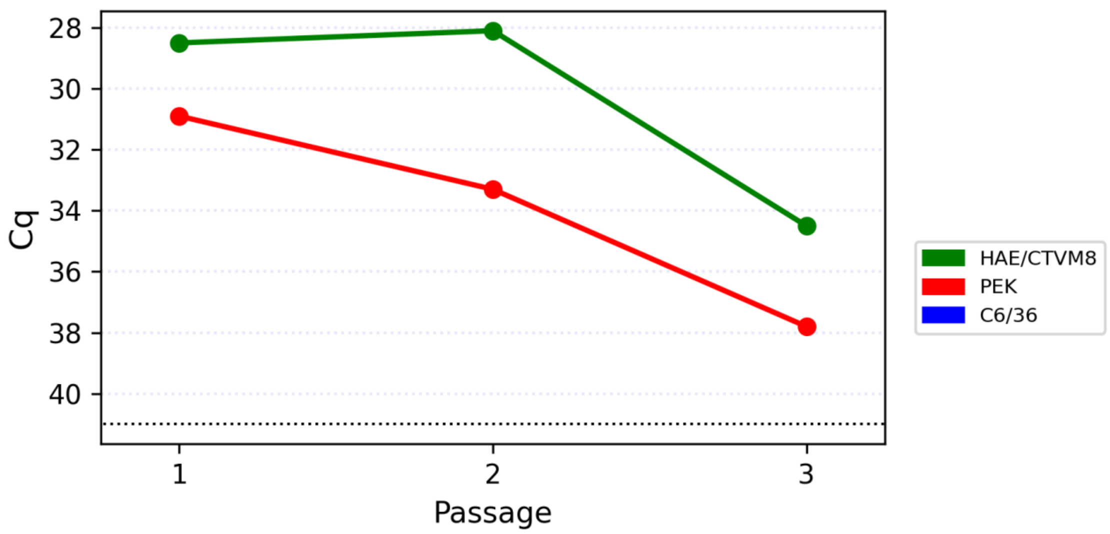

Figure S3. Dynamic of the real-time quantification cycle (Cq) during passages of the Kamenushka Hybomitra narna-like virus. Black dotted line represents qPCR detection limit (41 cycles).

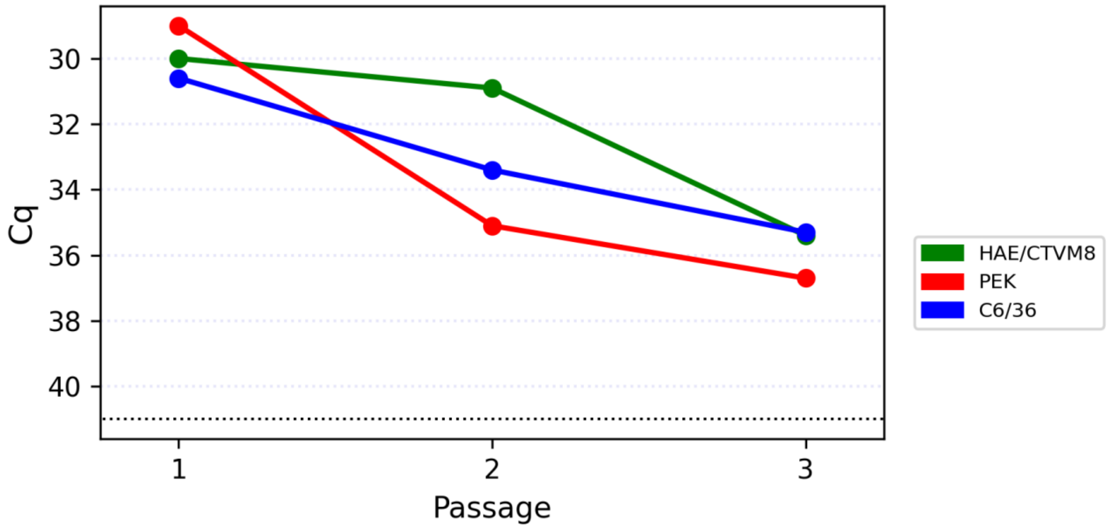

Figure S4. Dynamic of the real-time quantification cycle (Cq) during passages of the Medvezhye pound Haematopota permuto-like virus. Black dotted line represents qPCR detection limit (41 cycles).

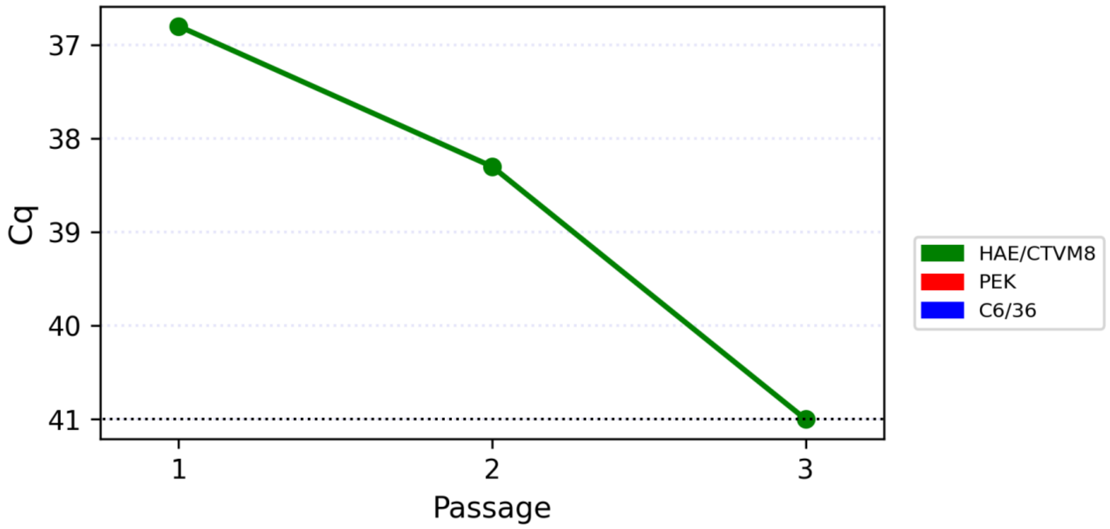

Figure S5. Dynamic of the real-time quantification cycle (Cq) during passages of the Polka Haematopota nora-like virus. Black dotted line represents qPCR detection limit (41 cycles).

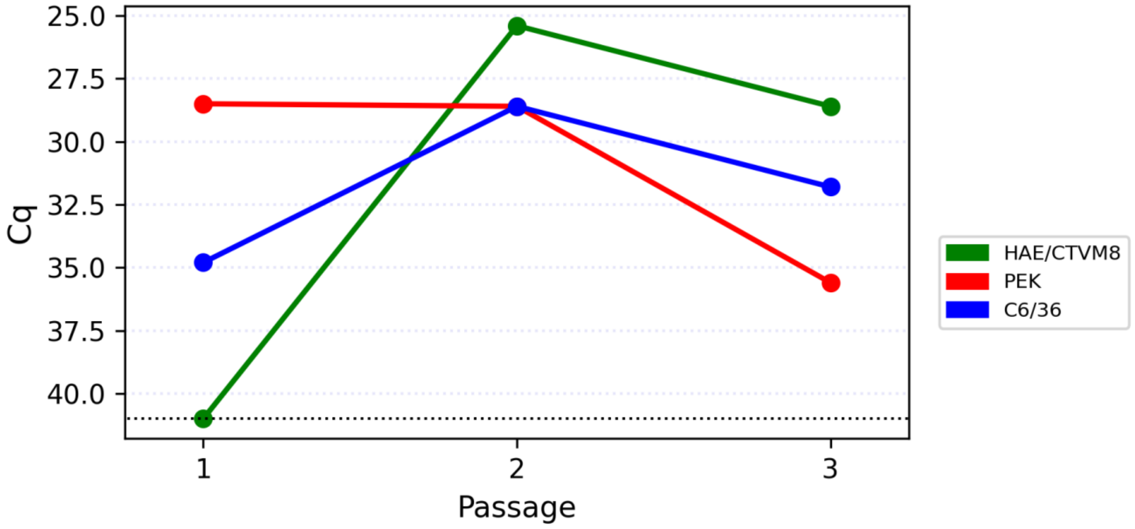

Figure S6. Dynamic of the real-time quantification cycle (Cq) during passages of the Big Soux River virus (strain Medvezhye). Black dotted line represents qPCR detection limit (41 cycles).

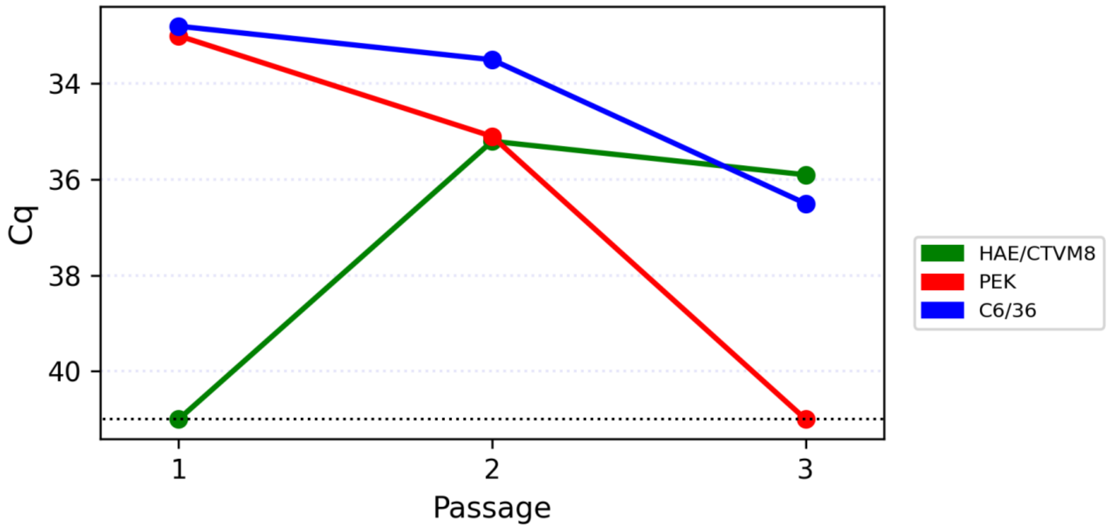

Figure S7. Dynamic of the real-time quantification cycle (Cq) during passages of the Melisia Chrysops solemo-like virus. Black dotted line represents qPCR detection limit (41 cycles).

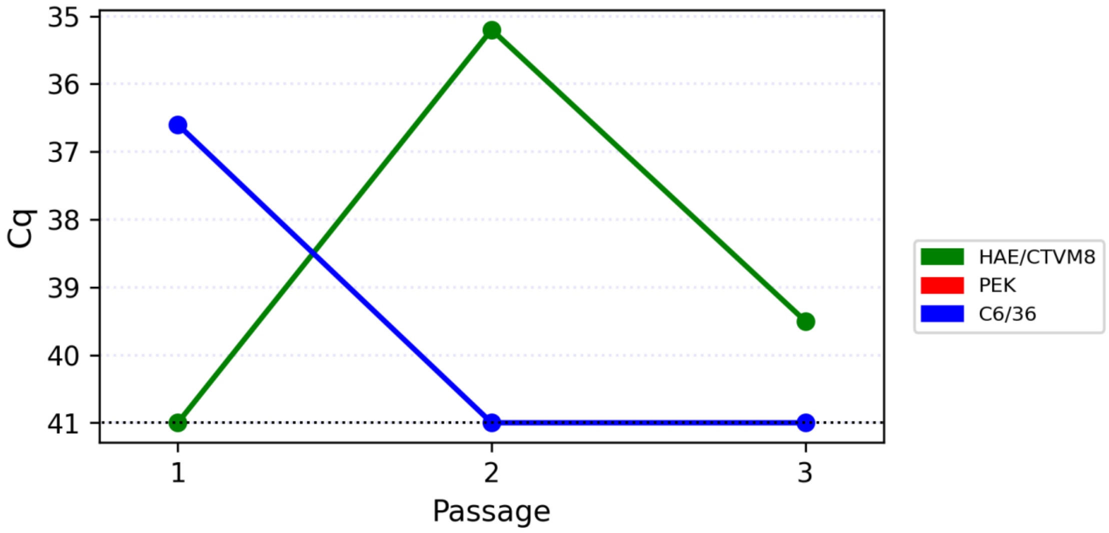

Figure S8. Dynamic of the real-time quantification cycle (Cq) during passages of the Polka Chrysops solemo-like virus. Black dotted line represents qPCR detection limit (41 cycles).

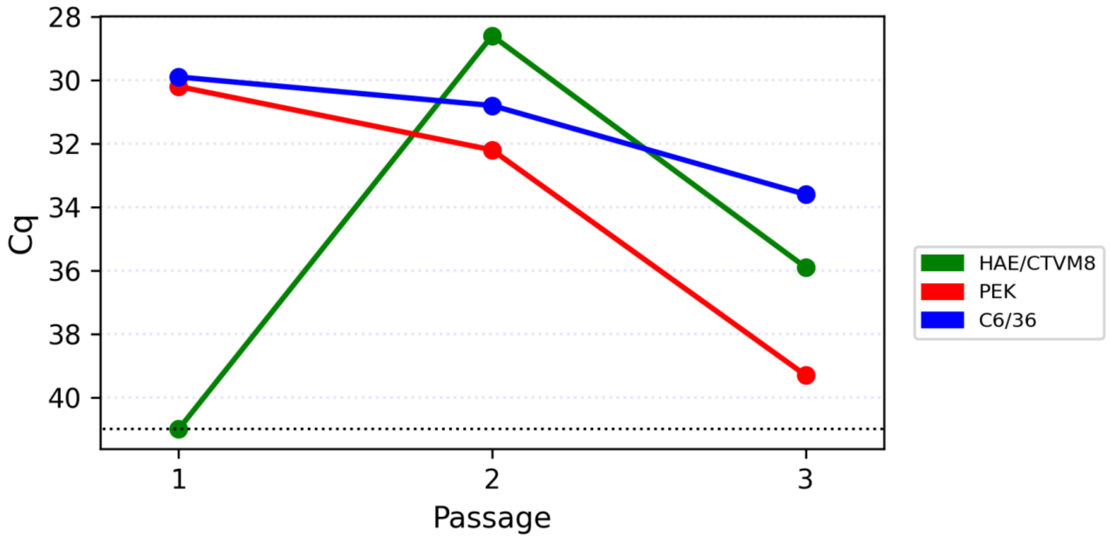

Figure S9. Dynamic of the real-time quantification cycle (Cq) during passages of the Medvezhye Chrysops narna-like virus 2. Black dotted line represents qPCR detection limit (41 cycles).

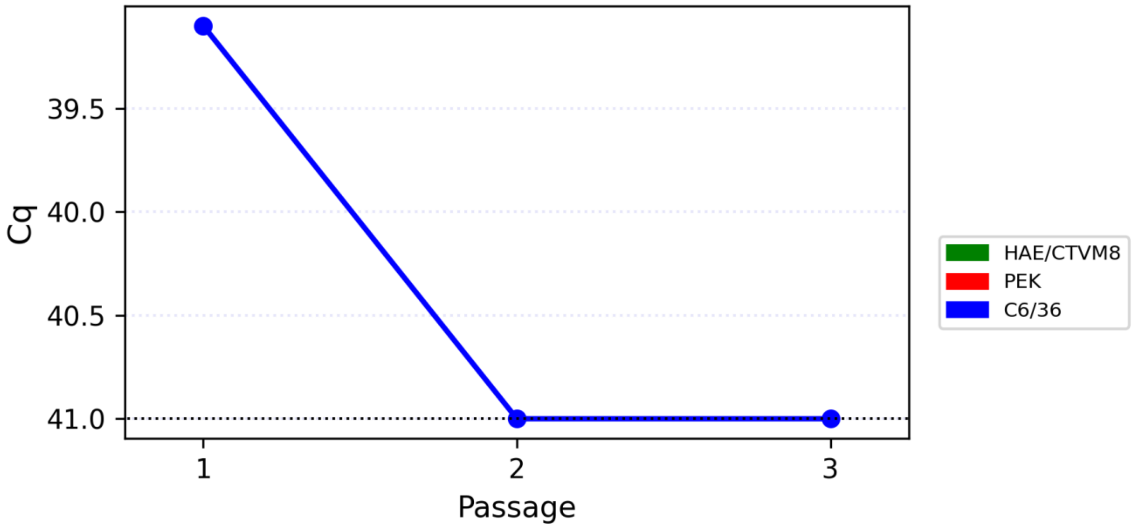

Figure S10. Dynamic of the real-time quantification cycle (Cq) during passages of the Medvezhye Chrysops rhabdo-like virus. Black dotted line represents qPCR detection limit (41 cycles).

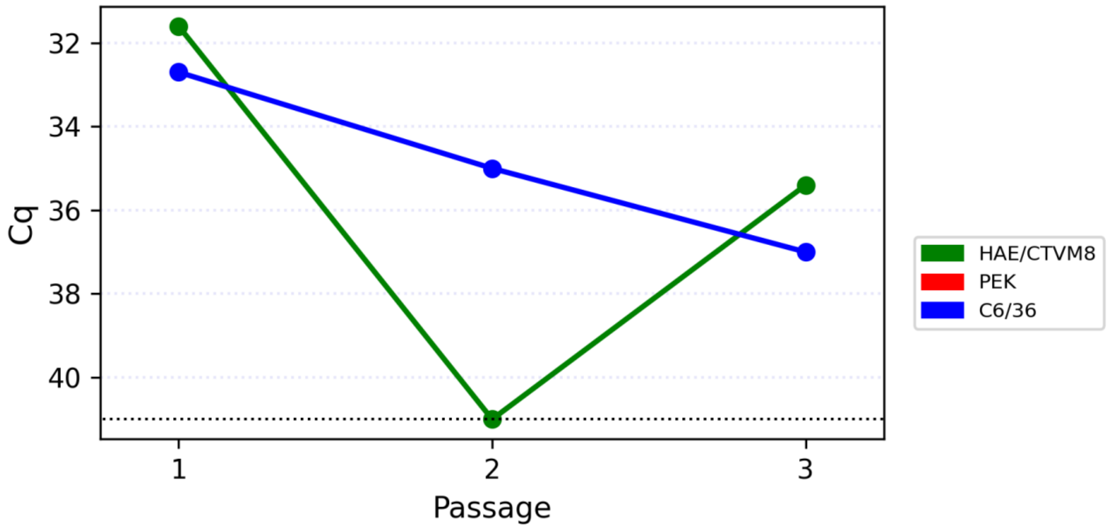

Figure S11. Dynamic of the real-time quantification cycle (Cq) during passages of the Istie Chrysops solemo-like virus. Black dotted line represents qPCR detection limit (41 cycles).

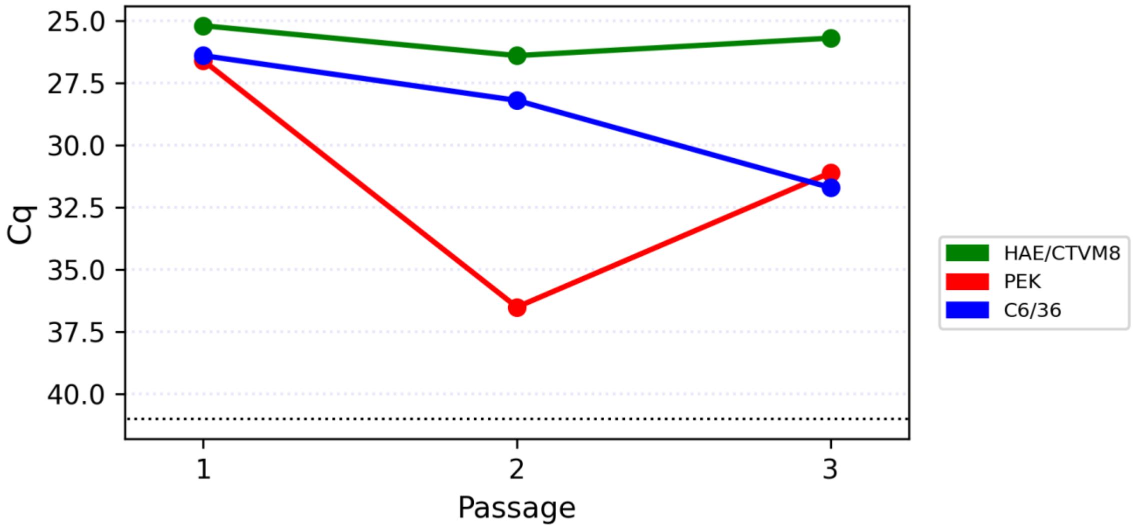

Figure S12. Dynamic of the real-time quantification cycle (Cq) during passages of the Medvezhye Tabanus toti-like virus. Black dotted line represents qPCR detection limit (41 cycles).

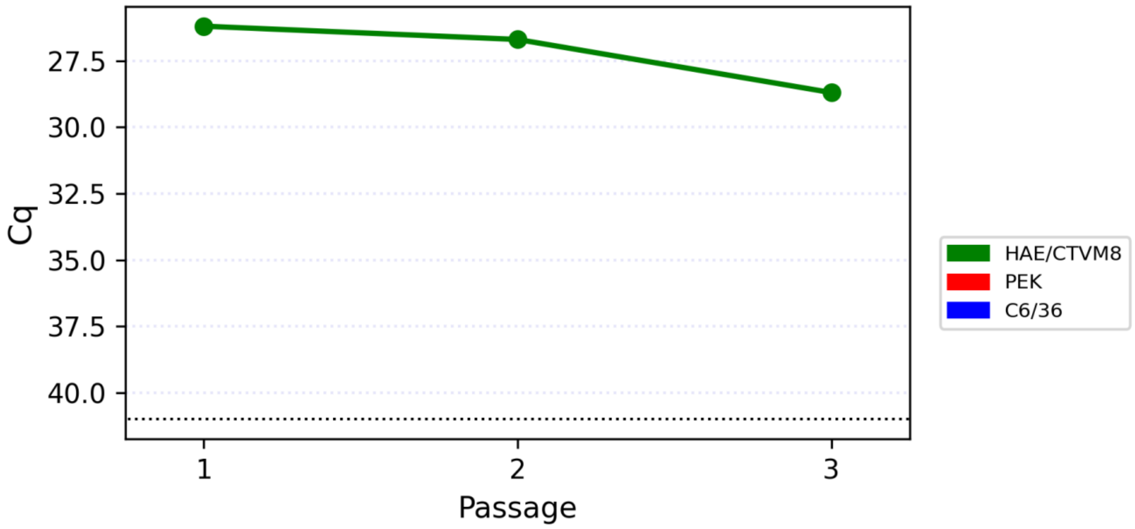

Figure S13. Dynamic of the real-time quantification cycle (Cq) during passages of the Komarovka hybomitra solemo-like virus. Black dotted line represents qPCR detection limit (41 cycles).

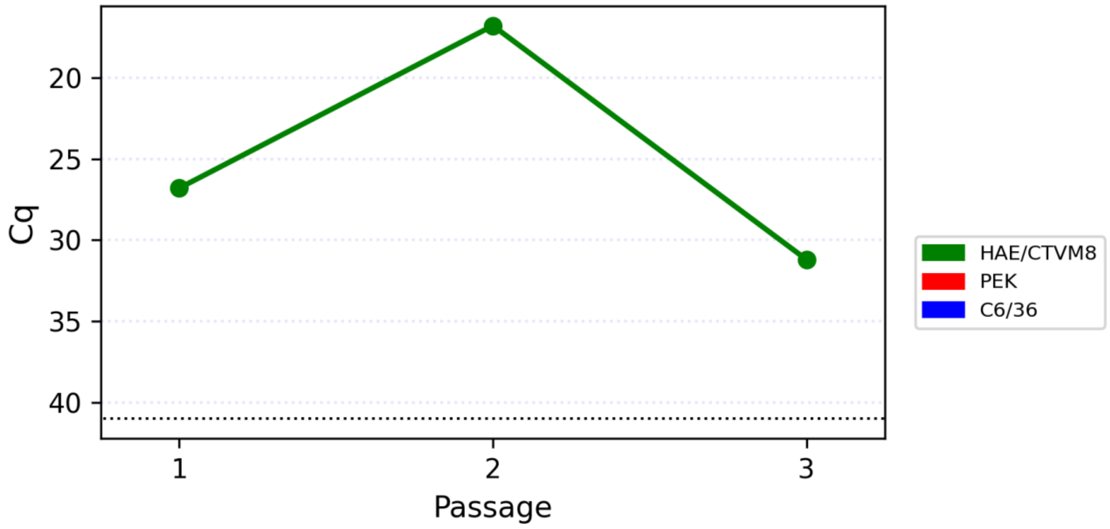

Figure S14. Dynamic of the real-time quantification cycle (Cq) during passages of the Barsukovka Hybomitra ifla-like virus. Black dotted line represents qPCR detection limit (41 cycles).
